# Preemptive Surgical Strategies for Impending Tracheo-Innominate Artery Fistula: A Case Series of Three High-Risk Patients

**DOI:** 10.3400/avd.cr.25-00154

**Published:** 2026-05-16

**Authors:** Yotaro Mori, Tomoki Nishimura, Noriyuki Takashima, Tomoaki Suzuki

**Affiliations:** Division of Cardiovascular Surgery, Department of Surgery, Shiga University of Medical Science, Otsu, Shiga, Japan

**Keywords:** tracheo-innominate artery fistula, preemptive surgery, tracheostomy

## Abstract

Tracheo-innominate artery fistula (TIF) is a rare but catastrophic complication of tracheostomy with extremely high mortality after rupture. No established guidelines exist for preemptive intervention in impending TIF. We report 3 high-risk patients with objective imaging and/or bronchoscopic warning signs who underwent individualized preemptive surgical management. Risk assessment included computed tomography or magnetic resonance imaging, bronchoscopy, and intraoperative cerebral perfusion monitoring during temporary innominate artery clamping. Strategies included muscle interposition, ligation, or intrathoracic bypass reconstruction. All patients recovered without neurological complications, and no TIF occurred during follow-up. Preemptive intervention may be considered only in carefully selected patients with objective warning signs.

## Introduction

Tracheo-innominate artery fistula (TIF) is an uncommon but catastrophic complication of tracheostomy, with an incidence of <1%.^1)^ Once bleeding occurs, the mortality rate exceeds 50%.^2)^ TIF usually results from mechanical erosion of the innominate artery by a tracheostomy tube.

Preventive strategies for TIF are important because outcomes become dismal once it develops. However, standardized indications and optimal surgical methods remain controversial.^3–5)^ Reported risk factors include a short neck, severe scoliosis, prior bleeding, and radiological evidence of tracheoinnominate artery proximity.^1,6–9)^ Here, we present cases of 3 high-risk patients who underwent preemptive surgical intervention, with surgical planning based on anatomical and intraoperative hemodynamic assessments.

## Case Report

### Case 1

**Fig. 1:**
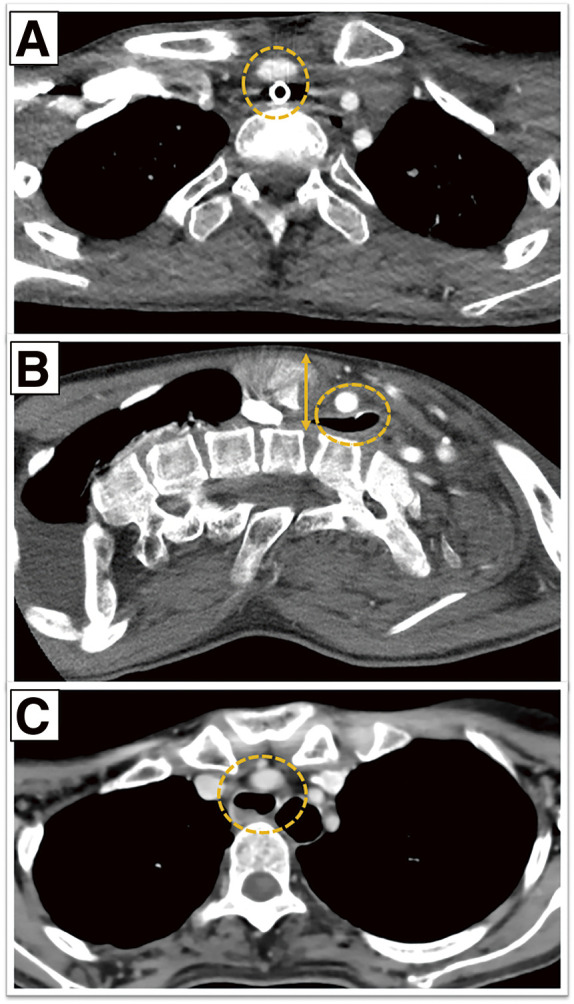
Preoperative contrast-enhanced computed tomography (CT) demonstrating impending tracheo-innominate artery fistula. (**A**) Axial view showing direct contact between the tracheostomy tube and the innominate artery (dashed circle). (**B**) Sagittal reconstruction demonstrating extremely narrow anteroposterior mediastinal space (18 mm) between the posterior surface of the sternum and the anterior surface of the cervical vertebra (arrow and dashed circle). (**C**) Axial CT image in another case showing close contact between the trachea and the innominate artery (dashed circle).

An 11-year-old male with neuronal ceroid lipofuscinosis and ventilator dependence presented with tracheostoma bleeding. Bronchoscopy revealed tracheal wall pulsation, and computed tomography (CT) showed close proximity between the tracheostomy tube and the innominate artery (**Fig. 1A**). Temporary clamping through a suprasternal incision reduced the right radial artery pressure from 90 to 50 mmHg, suggesting a risk of cerebral ischemia. Division was avoided, and the thymus and strap muscles were interposed. Postoperative recovery was uneventful, and no TIF occurred within 4 years of follow-up.

### Case 2

A 19-year-old male with severe scoliosis was referred to our hospital for tracheostomy. CT showed an 18-mm retrosternal distance with close proximity between the trachea and the innominate artery (**Fig. 1B**). Temporary clamping through a suprasternal incision with partial manubrial resection reduced the right radial pressure to 65 mmHg (left: 128 mmHg), indicating adequate collateral circulation. The innominate artery was ligated and divided. The patient recovered uneventfully.

### Case 3

**Fig. 2:**
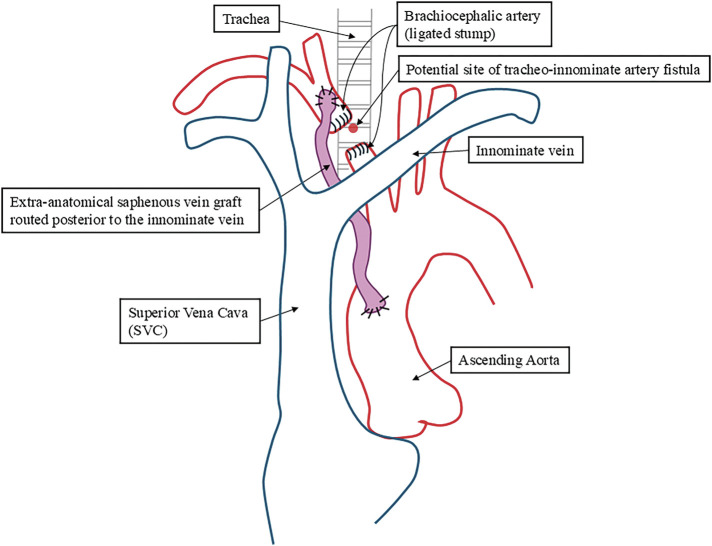
Schematic illustration of Case 3 showing intrathoracic bypass reconstruction from the ascending aorta to the innominate artery to prevent direct contact between the trachea and the innominate artery. Median sternotomy was selected because the anticipated closest point between the trachea and the innominate artery was located in the lower mediastinum.

A 42-year-old female with spinocerebellar degeneration presented with bleeding at the site of a tracheostomy. Bronchoscopy revealed friable tissue, and CT showed contact between the trachea and the innominate artery beneath the sternum (**Fig. 1C**). Median sternotomy was performed, and temporary clamping reduced the radial pressure to 40 mmHg, necessitating revascularization. A bypass graft from the ascending aorta to the innominate artery was constructed using the great saphenous vein. Graft patency was confirmed, and the patient recovered uneventfully (**Fig. 2**).

## Discussion

TIF is a rare but catastrophic complication of tracheostomy, with reported perioperative mortality exceeding 50%.^1,3)^ Although its overall incidence is low, once rupture occurs, survival is extremely limited even with immediate intervention.

Currently, no established guidelines exist regarding preemptive intervention for impending TIF. Therefore, surgical indications were determined on a case-by-case basis, integrating imaging findings, bronchoscopic evaluation, and failure of conservative management. Based on previous reports, recognized risk factors include anatomical proximity between the trachea and innominate artery, scoliosis, short neck, and prior bleeding episodes.^1,6–9)^ In our practice, preemptive surgery was considered only in highly selected patients with long-term tracheostomy who demonstrated close contact or severe narrowing between the tracheostomy tube and the innominate artery on CT. Previous reports have suggested that an anteroposterior mediastinal distance of less than 20 mm between the posterior surface of the sternum and the anterior surface of the cervical vertebra is associated with an increased risk of TIF.^6,7)^ Bronchoscopic findings, such as pulsatile granulation tissue or bleeding from tracheal granulation tissue, were regarded as sentinel signs of impending TIF.^3,7,8)^

In our series, objective high-risk findings were present in all patients: bronchoscopic abnormalities in Cases 1 and 3, and an extremely narrow anteroposterior mediastinal space (18 mm) between the posterior surface of the sternum and the anterior surface of the cervical vertebra on CT in Case 2. Importantly, treatment decisions were made through multidisciplinary conferences involving cardiovascular surgeons, pediatricians, and otolaryngologists, with careful consideration of surgical risks, neurological complications, and alternative conservative strategies.

Although this strategy may be described as prophylactic, all cases in this series represented impending TIF rather than routine prophylaxis. Surgical intervention was undertaken as preemptive rescue in highly selected circumstances based on objective warning signs.

Ethical considerations were central to decision-making. Nonoperative strategies, including tube repositioning and close radiologic or endoscopic surveillance, were considered in every case. Informed consent was obtained from patients’ families after a detailed explanation of neurological risks, the uncertainty of benefit, and alternative management options.

Preoperative assessment of cerebral collateral circulation was performed using magnetic resonance imaging in Case 1 and CT angiography in Cases 2 and 3.

In all patients, preoperative imaging studies confirmed that the circle of Willis was anatomically preserved. However, preoperative imaging alone was considered insufficient to determine the safety of innominate artery division. Therefore, the final decision regarding division or reconstruction was based primarily on intraoperative hemodynamic assessment during temporary occlusion. Previous reports have suggested that cerebral circulation can be maintained without revascularization when the stump pressure of the right common carotid artery during temporary occlusion of the innominate artery is ≥50–60 mmHg.^10,11)^ Because direct measurement of carotid stump pressure was not feasible in our cases, right radial arterial pressure was used as a practical surrogate marker. Based on these reports, we adopted a right radial arterial pressure of ≥60 mmHg during temporary occlusion as a practical criterion for determining whether revascularization was necessary.

Regional cerebral oxygen saturation (rSO_2_) monitoring using near-infrared spectroscopy was routinely employed intraoperatively; however, quantitative data could not be retrieved for retrospective analysis. Cerebral perfusion during temporary occlusion was primarily assessed based on the right radial arterial pressure, while rSO_2_ monitoring served as a supplementary indicator. No postoperative neurological complications were observed in any patient.

The surgical strategy differed among cases according to anatomical configuration and intraoperative feasibility. In Case 3, the anticipated closest point between the innominate artery and trachea was located more caudally than in Case 1 because the patient was relatively older. Furthermore, median sternotomy was the only feasible surgical approach. Therefore, intrathoracic bypass reconstruction was considered the most reliable strategy to ensure stable cerebral perfusion. In contrast, in Case 1, muscle interposition was selected to separate the trachea and the innominate artery without vascular reconstruction. Temporary clamping reduced the right radial arterial pressure to 50 mmHg, suggesting that simple ligation of the innominate artery could compromise cerebral perfusion. Although arterial division with revascularization was technically feasible, this strategy was avoided because the surgical field was located in close proximity to the tracheostomy site, raising concerns about potential infection. Therefore, muscle interposition was selected as a less invasive strategy to separate the trachea and the innominate artery without arterial division. Muscle interposition has been reported as a preventive strategy to separate the trachea from the innominate artery in patients at risk of TIF.^8,9)^ Although long-term durability remains uncertain due to the rarity of this procedure, no recurrence was observed during the follow-up period in this case.

During the past decade at our institution, only 4 patients were identified with TIF-related pathology. Of these, 3 were managed as impending TIF with preemptive intervention, whereas 1 presented with rupture complicated by cardiac arrest requiring emergent salvage surgery. This experience underscores the catastrophic nature of established TIF and supports consideration of preemptive intervention in carefully selected impending cases.

This study is limited by its retrospective design and small sample size, and selection bias is unavoidable. No denominator population of conservatively managed high-risk patients is available. Therefore, these findings should not be generalized, and preemptive intervention should be considered only in exceptional cases with objective signs of impending TIF.

Preemptive surgical intervention should be reserved exclusively for patients with objective radiologic and bronchoscopic warning signs and should not be applied as routine prophylaxis.

## Conclusion

Preemptive intervention for TIF should not be generalized. However, in highly selected patients with objective imaging and bronchoscopic warning signs of impending TIF, individualized surgical strategies based on anatomical configuration and cerebral perfusion assessment may provide a viable preemptive option to prevent catastrophic rupture.

## Declarations

### Data availability

Data are available from the corresponding author on reasonable request.
